# Estimation of the octanol-water distribution coefficient of basic compounds by a cationic microemulsion electrokinetic chromatography system

**DOI:** 10.5599/admet.760

**Published:** 2020-03-04

**Authors:** Alejandro Fernández-Pumarega, Belén Martín-Sanz, Susana Amézqueta, Elisabet Fuguet, Martí Rosés

**Affiliations:** 1 Departament d’Enginyeria Química i Química Analítica and Institut de Biomedicina (IBUB), Facultat de Química, Universitat de Barcelona, Martí i Franquès 1-11, 08028, Barcelona, Spain; 2 Serra Húnter Programme. Generalitat de Catalunya. Spain; § Editorial Board member

**Keywords:** Lipophilicity, log *P*_o/w_, log *D*_o/w_, MEEKC, ionized bases, solvation parameter model, microemulsion, system surrogation

## Abstract

The octanol-water partition coefficient (P_o/w_), or the octanol-water distribution coefficient (D_o/w_) for ionized compounds, is a key parameter in the drug development process. In a previous work, this parameter was estimated through the retention factor measurements in a sodium dodecyl sulfate (SDS) - microemulsion electrokinetic chromatography (MEEKC) system for acidic compounds. Nonetheless, when ionized basic compounds were analyzed, undesirable ion pairs were formed with the anionic surfactant and avoided a good estimation of log D_o/w_. For this reason, an alternative MEEKC system based on a cationic surfactant has been evaluated to estimate P_o/w_ or D_o/w_ of neutral compounds and ionized bases. To this end, it has been characterized through the solvation parameter model (SPM) and compared to the octanol-water partition system. Results pointed out that both systems show a similar partition behavior. Hence, the log P_o/w_ of a set of neutral compounds has been successfully correlated against the logarithm of the retention factor (log k) determined in this MEEKC system. Then, the log D_o/w_ of 6 model bases have been estimated at different pH values and they have been compared to data from the literature, determined by the reference shake-flask and potentiometric methods. Good agreement has been observed between the literature and the estimated values when the base is neutral or partially ionized (up to 99% of ionization).

## Introduction

Chromatographic systems based on different techniques such as high performance liquid chromatography (HPLC) and electrokinetic chromatography (EKC) have been widely used to determine biopartitioning properties [1–6]. In these systems, the compounds experience a partition between an aqueous phase and a stationary phase, for HPLC systems, or a pseudoestationary phase, for EKC systems, similar to the partition that compounds experience in a biological system. Therefore, if the partition in the chromatographic system and in the biological one is similar enough, it is possible to estimate the biological property through a correlation like Eq. 1:


(1)

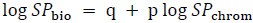



where *SP_bio_* is the solute biological property, *SP_chrom_* the solute physicochemical property (generally the retention factor, *k*), and “q” and “p” are the intercept and the slope of the resulting correlation between both parameters, respectively.

The lipophilicity of compounds is a property of high interest in biological processes due to its direct relationship with membrane permeability. Lipophilicity is usually evaluated through the octanol-water partition coefficient (*P*_o/w_). The direct method to determine this parameter is the shake-flask method. However, it is a tedious procedure and it is not fully automated. For this reason, the estimation of *P*_o/w_ through high-throughput systems such as chromatographic ones is of great interest.

In the previous works [3–6], Eq. 1 was applied to the determination of the octanol-water partition coefficient of neutral solutes and the distribution coefficient of partially ionized acidic compounds (*D*_o/w_) from microemulsion electrokinetic chromatography (MEEKC) measurements. A MEEKC system based on a negatively charged microemulsion (ME) constituted of heptane, 1-butanol, and sodium dodecyl sulfate (SDS) was used. The aim of the present study is to evaluate the applicability of MEEKC measurements to estimate the log *P*_o/w_ and log *D*_o/w_ of ionized basic compounds, which are positively charged in their ionized form.

However, the SDS-MEEKC system cannot be used for basic compounds since the formation of ion pairs between the surfactant, negatively charged, and the protonated base, positively charged has been observed. Thus, the retention factor would be altered leading to wrong log *D*_o/w_ estimated values. In this work, the replacement of the anionic surfactant (SDS) by a cationic one (tetradecyltrimethylammonium bromide, TTAB) is tested to solve this problem. So, as the only role of the surfactant is to stabilize the oil droplets, we think that the TTAB-MEEKC system should be able to estimate the *P*_o/w_ or *D*_o/w_ of basic solutes as well as the SDS-MEEKC does for acidic solutes. Actually, Ishihama *et al.* [7] evaluated 3 different surfactants and showed that neither the ionic groups nor the hydrocarbon chain lengths affected the selectivity of the ME for neutral compounds. Thus, the aim of the present work is to test this potential ability of the TTAB-MEEKC system.

## Theory

The retention factor of an ionized base can be calculated through Eq. 2 [8]:


(2)

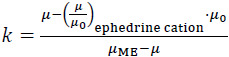



where, *μ* and *μ*_0_ are the electrophoretic mobility of the compound in MEEKC and in plain buffer (capillary zone electrophoresis, CZE), respectively. *μ_ME_* is the electrophoretic mobility of the ME marker in the MEEKC analysis, and *(μ*/*μ*_0_*)*_ephedrine cation_ is the viscosity correction factor (whose value is 0.84). This correction needs to be introduced since two solutions with quite different viscosities (*ƞ*) are employed in *k* calculation, and *μ* is inversely related to *ƞ* [9]. Therefore, the mobility in CZE is corrected to make it equivalent to the one measured in MEEKC. Ephedrine has been selected because it is a small and polar compound that is not retained in the chromatographic system when it is fully ionized. So, (*μ/μ*_0_)*_ephedrine cation_* results from the ratio of mobilities of the ephedrine cation measured in MEEKC and CZE modes.

*μ* values can be obtained from:


(3)

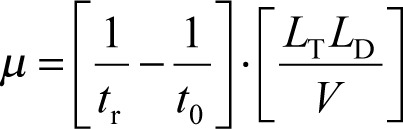



where, *t*_r_ and *t*_0_ are the elution times of the compound and the electroosmotic flow marker, respectively. *L*_T_ and *L*_D_ are the total and the effective length of the capillary, respectively, and *V* is the voltage applied during the separation.

Similarly to acidic solutes [10], the retention factor of a monoprotic basic compound in a MEEKC system varies with the pH of the media through:


(4)

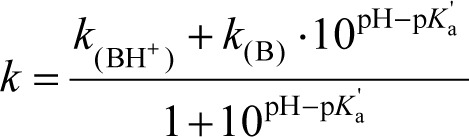



where *k*_(B)_ and *k*_(BH+)_ are the retention factors of the neutral and the fully ionized base, respectively, and the p*K*_a_’ is the apparent acidity constant of the base.

## Experimental

### Equipment

All the analysis were performed with a capillary electrophoresis (CE) 7100 equipped with a UV diode array detector from Agilent technologies (Santa Clara, CA, USA). The fused-silica capillaries employed were from Polymicro Technologies (Phoenix, AZ, USA). The capillaries used had a 50 μm internal diameter.

Water was purified by a Milli-Q plus system from Millipore (Burlington, MA, USA) with a resistivity of 18.2 MΩ·cm. To determine the pH of the solutions a pH-meter GLP 22 from Crison (Barcelona, Spain) was used.

### Reagents

Hydrochloric acid (1N Tritisol^TM^), and sodium hydroxide (0.5N Tritisol^TM^) were acquired from Merck (Darmstadt, Germany). Methanol (HPLC-grade) was obtained from Thermo Fisher Scientific (Waltham, MA, USA). Heptane (99%), dodecanophenone (98%), TTAB (>99%), 1-butanol (≥99.7%), 2-[Bis(2-hydroxyethyl)amino]-2-(hydroxymethyl)propane-1,3diol (Bis-tris) (>99%), and sodium phosphate dodecahydrate (>98%) were purchased from Sigma-Aldrich (St. Louis, MO, USA). Disodium hydrogen phosphate (99.5%) was from Baker (Phillipsburg, NJ, USA).

Test compounds with high purities were supplied from different manufacturers: Sigma-Aldrich, Merck, Baker, Carlo Erba (Milan, Italy), Fluka (St. Louis, MO, USA), Riedel-de Häen (Seelze, Germany), and Scharlab (Barcelona, Spain).

### Analysis conditions

#### Buffer preparation

To prepare the buffers at pH 5.4 and pH 7.0, the pH of an aliquot of a 0.2 M bis-tris solution previously protonated with HCl was adjusted with NaOH, while to prepare the buffer at pH 11.4 a mixture of a 0.2 M disodium hydrogen phosphate solution and a 0.2 M sodium phosphate dodecahydrate solution was used. All the buffers were prepared maintaining the ionic strength at 0.05 M.

#### ME preparation

To prepare the ME 1.70, g of TTAB were dissolved in around 70 mL of the corresponding buffer solution. Then, 8.15 mL of 1-butanol were added, finishing by the addition of 1.15 mL of heptane. The co-surfactant and the oil were added under continuous magnetic stirring, and if the solution remained turbid, it was sonicated until clarification [4]. Finally, buffer was added up to a total volume of 100 mL. The concentrations of each component with respect the total volume of the ME were: 1.70% (w/v) TTAB, 8.15% (v/v) 1-butanol, and 1.15% (v/v) heptane.

#### Instrumental parameters

All the measurements were performed keeping the temperature at 25 °C and using a fused-silica capillary with an effective length of 30 cm and a total length of 38.5 cm. The voltage applied in MEEKC measurements was negative and between -11.5 and -14 kV. Regarding the internal pressure applied, it ranged between 0 and 25 mbar. The analysis conditions were different, depending on the pH, to obtain the best electrophoretic window possible. In the case of CZE measurements, the analyses were performed employing positive voltages.

Most compounds were dissolved at 200 mg·L^-1^ in a 9:1 ME:methanol mixture in the MEEKC analysis and in a 9:1 buffer:methanol mixture in the CZE analysis. Solutes with low UV absorbance were analyzed at a concentration of 10% (v/v).

The injection of the compounds was performed applying during 5 s an internal pressure of 50 mbar. The detection of the solutes was performed at λ=200, 214 or 254 nm (depending on the absorbance profile of the compounds). The ME marker used was dodecanophenone (200 mg·L^-1^, detected at λ = 254 nm), while the electroosmotic flow marker was methanol (10% v/v, detected at *λ* = 200 nm) [11].

### Comparison of systems characterized by the solvation parameter model

The TTAB-MEEKC system has been characterized through the solvation parameter model (SPM) to check its suitability to surrogate the octanol-water partition system [12]. SPM relates a free energy solvation property (in this work the log *P*_o/w_ or the log *k*) to five different solute descriptors. The equation resulting from the characterization of a system through the SPM model is shown below:


(5)

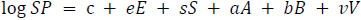



where SP is the solute property in a given partitioning system; *E*, *S*, *A*, *B* and *V* are the Abraham solute descriptors (which are specific for each compound), and the coefficients *e*, *s*, *a*, *b* and *v* provide the characteristics of the partition system. *E* is the excess molar refraction, *S* the solute dipolarity/polarizability, *A* and *B* are the solute hydrogen-bond acidity and basicity descriptors, respectively, and *V* the McGowan’s volume of the molecule. Each of the system coefficients are complementary to their respective solute descriptor. The system coefficients can be calculated by a multiple linear regression between the depending solute property of a set of representative neutral compounds and their solute descriptors [13].

The similarity between different systems can be determined by comparing the coefficients resulting from the characterization of the systems by the SPM. Lazaro, *et al.* [14] proposed the distance parameter *d* to compare two different systems (i and j) through their normalized SPM coefficients (Eqs. 6-10).


(6)

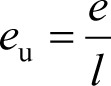




(7)

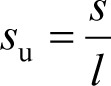




(8)

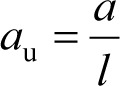




(9)

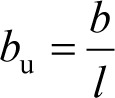




(10)

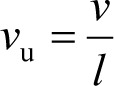



where *e*_u_, *s*_u_, *a*_u_, *b*_u_, *v*_u_ are the normalized coefficients (or coefficients of the unitary vector) and *l* is the length of the coefficients vector; which is calculated as follows:


(11)

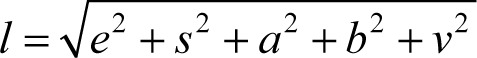



then, the *d* parameter is calculated by Eq. 12.


(12)

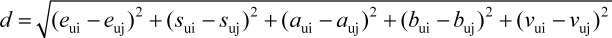



The smaller the *d* parameter, the more similar will be the systems compared. As a practical convention, if the distance between the systems compared is below 0.25, it can be concluded that these systems are analogous [14].

Moreover, the precision of the correlation (Eq. 13) between log *P*_o/w_ and log *k* in the TTAB-MEEKC system has been estimated following the approach published elsewhere [15].

The overall precision of the correlation (*SD*_corr_^2^) can be estimated by the sum of three different contributions:


(13)

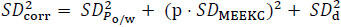



*SD*_Po/w_ and *SD*_MEEKC_ are the standard deviations (SD) obtained from the characterization through the SPM of the octanol-water partition system and the TTAB-MEEKC system, respectively. *SD*_d_ and “p” are the SD and the slope obtained from the correlation of the log *P*_o/w_ and log *k* values of a set of representative solutes. The values of the parameters were calculated using the equations resulting from the SPM characterization and the descriptors of the solutes. By using these equations, *SD*_Po/w_ and *SD*_MEEKC_ are equal to 0. So, the SD of this correlation can be attributed uniquely to the dissimilarity between the systems.

### Data analysis

Table Curve 2D from Systat Software Inc. (San Jose, CA, USA) was employed to obtain the log *D*_o/w_-pH profiles of the basic compounds. Data calculations were performed using Excel 2010 from Microsoft (Redmond, WA, USA).

## Results and Discussion

### Suitability of the TTAB-MEEKC system to estimate log P_o/w_ values

#### Characterization of the MEEKC system by the SPM

The TTAB-MEEKC system has been characterized through the SPM in order to later evaluate its similarity with the octanol-water partition system. To obtain accurate solvation coefficients of the model, representative analytes have been selected to characterize the system. In 2001, Fuguet *et al.* [13] proposed 71 compounds from a 2975 solute data base, selected according to their solute-solvent interactions, as a good set to characterize chromatographic systems by the SPM.

The retention factors of the proposed set of compounds in the TTAB-MEEKC system have been measured at pH 7.0, where all compounds are neutral. During the characterization, some of the compounds have been discarded since they are not lipophilic enough and elute with the EOF marker (propan-1,3-diol, butan-1,4-diol, and pentan-1,5-diol), they are too lipophilic and they co-elute with the ME marker (butylbenzene, and α-pinene), or they present other experimental troubles in the TTAB-MEEKC system (myrcene, pentan-1-ol and pentan-3-ol). The retention factor of neutral compounds can be calculated using Eq. 2, being *μ_0_* equal to 0 as they are uncharged. Table 1 lists the compounds used in the characterization together with their solute descriptors, and the log *k* in the TTAB-MEEKC system. Moreover, the log *P_o/w_* values of each compound reported in the literature are also listed [16–30].

To obtain the SPM coefficients of the TTAB-MEEKC system, butan-1-ol, and thiourea were considered as outliers and discarded, as they presented standard residues higher than 2.5. Estradiol had a standard residue slightly greater than 2.5. However, it was not considered as outlier because discarding it had no big influence on the result.

The parameters and statistics (F, Fisher’s F parameter; SD, standard deviation; R^2^, determination coefficient; n, number of compounds) resulting from the system characterization are:


(14)

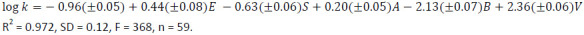



The large values of the coefficients *b* and *v* show that the hydrogen bond basicity and the volume of the solute are the more important parameters. The *v* coefficient is positive, meaning that it is easier to form a cavity to place the solute in the ME phase than in the aqueous one. A negative *b* coefficient is obtained indicating a higher hydrogen bonding acidity of the aqueous phase with respect to the ME phase. The *e* coefficient is positive, so the ME is more polarizable than the aqueous phase. The negative value of the *s* coefficient indicates that the ME system is less dipolar than the aqueous phase. The coefficient *a* is close to zero, showing that hydrogen bonding basicities of ME and aqueous phases are similar, and the variation of solute hydrogen bond acidity has a small effect on the system.

#### Model comparison

The TTAB-MEEKC system has been compared to the octanol-water partition system and the SDS-MEEKC system. The last system was used in other research studies to estimate the log *P*_o/w_ of ionizable acids [5, 6].

The normalized coefficients obtained from the characterization of the TTAB-MEEKC, the octanol-water partition [31], and the SDS-MEEKC [3] (calculated using Eqs. 6-11), and the *d* distance parameter (Eq. 12) are summarized in Table 2.

The normalized coefficients of the three systems are very similar, being for all of them the *b* and *v* coefficients the most important ones. Furthermore, the *d* parameter between TTAB-MEEKC and octanol-water partition is very small (0.06), meaning that the TTAB-MEEKC system is a good approximation to surrogate the octanol-water partition one, and consequently, lipophilicity. The *d* parameter obtained between TTAB-MEEKC and SDS-MEEKC is also small (0.11), therefore TTAB is a good substitute of SDS for log *P*_o/w_ determination.

The chromatographic precision of the correlation between log *k* in the TTAB-ME and the octanol-water partition has also been calculated. As explained before and elsewhere in the literature [15], the overall precision results can be estimated from a sum of three different contributions (*SD_P_*_o/w_*^2^*, *(*p·*SD*_MEEKC_)^2^, *SD*_d_^2^). Here, the largest contribution to the overall precision comes from the chromatographic data ((p·*SD*_MEEKC_)^2^ = 0.034) and the dissimilarity between the two systems (*SD*_d_^2^ = 0.013) is similar than the variance of the biopartitioning data (*SD_P_*_o/w_^2^ = 0.013). However, the calculated overall precision (*SD*_corr_^2^ = 0.061) is low. Therefore, the correlation obtained between the TTAB-MEEKC and octanol-water systems should be good, meaning that, the TTAB-MEEKC system is a good candidate to estimate satisfactorily the octanol/water lipophilicity of neutral compounds.

#### Correlation between log *P*_o/w_ and log *k*

**Figure 1.**
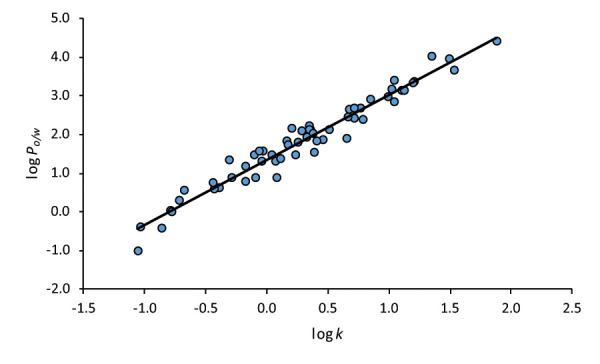
log *P*_o/w_ vs log *k* correlation with the compounds from Table 1 (Eq. 15).

A linear correlation between the log *P*_o/w_ and the log *k* of 58 solutes presented in Table 1 has been established. The correlation between log *P*_o/w_ and log *k* is plotted in Figure 1. The equation and statistics obtained are as follows:


(15)

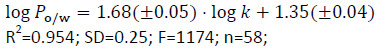



which is not significantly different from the log *P*_o/w_ – log *k* correlation already reported for the SDS-MEEKC system employing a set of neutral compounds (Eq. 16) [6].


(16)

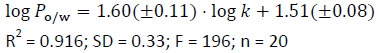



Good statistics have been obtained for the correlation: R^2^ value close to 1, and a small SD value of the regression, whereby the TTAB-MEEKC system has been proved to provide a good estimation of the log *P*_o/w_ of neutral compounds, as already pointed out through the model comparison tools. Therefore, Eq. 15 is going to be used as calibration curve for the estimation of *D*_o/w_ of ionized bases in the following section.

### log D_o/w_ estimation of ionized basic compounds.

With the aim to extent the applicability of the method to ionizable bases, the log *D*_o/w_ of six basic compounds (alprenolol, nadolol, oxprenolol, penbutolol, pindolol, and propranolol) have been estimated at different pH values, thus at different ionization degrees and compared to literature experimental values. All these compounds are used as pharmaceutical drugs and present a basic group with a p*K*_a_ inside the electrophoretic p*H* working range (2-12) [27].

**Figure 2.**
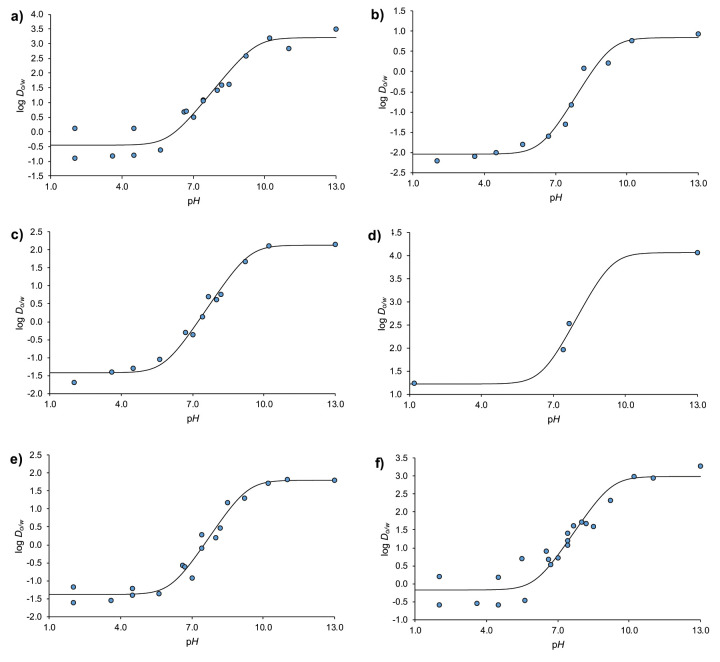
log *D*_o/w_ – p*H* profiles obtained adjusting Eq. 17 to the data from the literature (•): a) alprenolol, b) nadolol, c) oxprenolol, d) penbutolol, e) pindolol, f) propranolol.

Similarly to *k* (Eq. 4), the log *D*_o/w_ of ionized bases depends on the p*K*_a_’ of the base and the p*H* of the ME through:


(17)

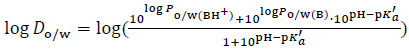



where log *P*_o/w(BH+)_ and log *P*_o/w(B)_ are the logarithms of the octanol–water partition coefficient of the fully ionized and neutral base, respectively. The log *D*_o/w_ – pH profiles for each of the model compounds have been calculated fitting Eq. 17 to the log *D*_o/w_ values determined at different values of pH taken from the literature [32–37]. The log *D*_o/w_ values compiled have been determined using the reference shake-flask, and the pH-metric titration methods. The profiles, the parameters and the statistics resulting from these fittings can be found in Figure 2 and Table 3.

Then, the *k*_(BH+)_ and *k*_(B)_ of the bases have been determined in the TTAB-MEEKC system at pH 5.4 and 11.4, respectively (Table 3). The retention factors and the p*K*_a_’ of each of the bases have been used in Eq. 4 to estimate *k* values at the pH of interest. Then, log *D*_o/w_ has been estimated through Eq. 15. The log *D*_o/w_ from the literature [32–37] and the estimated values are summarized in Table 4. The ionization degree (*α*_(BH+)_) of the bases at each pH value (calculated using Eq. 18) are also provided.


(18)

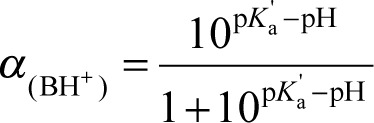



**Figure 3.**
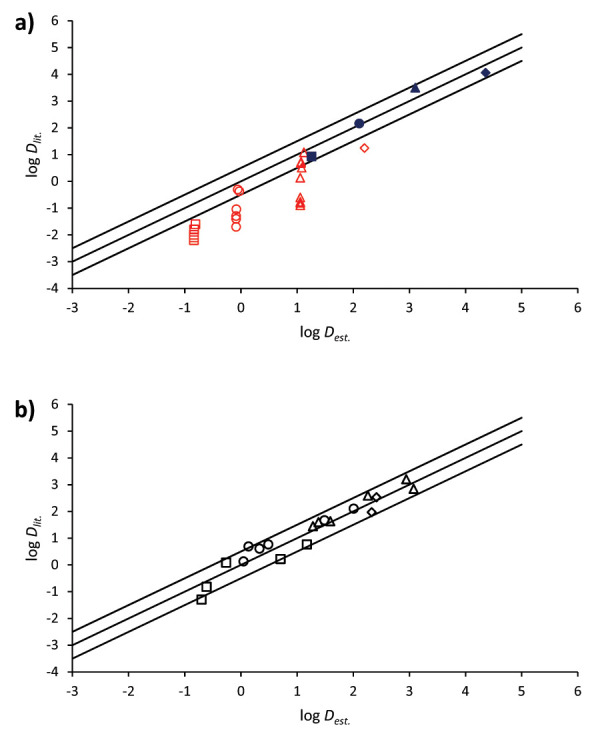
Correlation between the log Do/w from the literature (log Dlit.) and the log Do/w estimated through the present method (log Dest.) at different degrees of ionization. Alprenolol (▲), nadolol (■), oxprenolol (●), penbutolol (♦), pindolol (✖), and propranolol (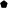). a) Comparison of the data when the bases are in their neutral form (0-1% of ionization, blue full symbol) or fully ionized (≥99% of ionization, red empty symbol). b) Comparison of the data when the bases are partially ionized (1-99% of ionization). In addition, a line with a slope of 1 and an intercept of 0, and two extra lines corresponding to ±2 SD of the calibration curve (Eq. 15) (which corresponds to the 95% confidence interval) are also shown.

Figure 3 compares the estimated and the experimental data from Table 4 for bases neutral or fully ionized (Figure 3A) or partially ionized, from 1 to 99% (Figure 3B). The same figure also represents a line with a slope of 1 and an intercept of 0, and two extra lines showing the 95% confidence interval (±2 SD) of the calibration curve for neutral compounds.

Analyzing the results from Figure 3 and Table 4, it can be concluded that, generally, accurate estimated log *D*_o/w_ values are obtained especially when the compound is in its neutral or partially ionized forms. The error obtained is similar to the one obtained for neutral compounds, as indicated by the error bars. Nonetheless, when the bases are highly or totally ionized (*α* ≈ 1) an overestimation of the parameter is observed. Note that when the compound is completely ionized the results are less comparable due to the high dependence of the log *P*_o/w(BH+)_ value with the solution medium (nature of counter-ions and concentrations as usually ion pairs are formed).

The results obtained in this work agree with the ones published before for acidic compounds when a SDS-MEEKC system was employed [6].

## Conclusions

The TTAB-MEEKC system characterized through the solvation parameter model shows great similarity with the octanol-water partition and the SDS-MEEKC systems. Thus, both ME-based systems can be used for log *P*_o/w_ estimation of neutral solutes.

A good correlation has been obtained between log *P*_o/w_ data and log k measured in the TTAB-MEEKC system for neutral compounds. Moreover, the applicability of the method has been widen to the estimation of log *D*_o/w_ of partially ionized bases. Accurate estimated values have been obtained when the compound is neutral or partially ionized (up to a 99% of ionization).

## Figures and Tables

**Table 1.** Abraham solute descriptors, log *k* and log *P_o/w_* of the chosen neutral analytes

Compound	*E*	*S*	*A*	*B*	*V*	log *k*	log *P*_o/w_^a)^
Propan-1-ol	0.236	0.42	0.37	0.48	0.5900	-0.71	0.30
Propan-2-ol	0.212	0.36	0.33	0.56	0.5900	-0.79	0.05
Butan-1-ol	0.224	0.42	0.37	0.48	0.7309	0.08	0.88
Pentan-1-ol	0.219	0.42	0.37	0.48	0.8718	-	1.56
Pentan-3-ol	0.218	0.36	0.33	0.56	0.8718	-	1.21
Propan-1,3-diol	0.397	0.91	0.77	0.85	0.6487	-	-1.04
Butan-1,4-diol	0.395	0.93	0.72	0.90	0.7860	-	-0.83
Pentan-1,5-diol	0.388	0.95	0.72	0.91	0.9305	-	-0.43
Thiourea	0.840	0.82	0.77	0.87	0.5696	-1.06	-1.02
Benzene	0.610	0.52	0.00	0.14	0.7164	0.36	2.13
Toluene	0.601	0.52	0.00	0.14	0.8573	0.77	2.69
Ethylbenzene	0.613	0.51	0.00	0.15	0.9982	1.10	3.15
Propylbenzene	0.604	0.50	0.00	0.15	1.1391	1.53	3.68
Butylbenzene	0.600	0.51	0.00	0.15	1.2800	-	4.38
*p*-Xylene	0.613	0.52	0.00	0.16	0.9982	1.12	3.15
Naphthalene	1.340	0.92	0.00	0.20	1.0854	1.21	3.37
Chlorobenzene	0.718	0.65	0.00	0.07	0.8388	0.85	2.90
Bromobenzene	0.882	0.73	0.00	0.09	0.8914	0.99	2.99
Anisole	0.708	0.75	0.00	0.29	0.9160	0.28	2.11
Benzaldehyde	0.820	1.00	0.00	0.39	0.8730	-0.10	1.47
Acetophenone	0.818	1.01	0.00	0.48	1.0139	-0.03	1.58
Propiophenone	0.804	0.95	0.00	0.51	1.1548	0.35	2.24
Butyrophenone	0.797	0.95	0.00	0.51	1.2957	0.68	2.65
Valerophenone	0.795	0.95	0.00	0.50	1.4366	1.05	3.40
Heptanophenone	0.720	0.95	0.00	0.50	1.7184	1.88	4.41
Benzophenone	1.447	1.50	0.00	0.50	1.4808	1.02	3.18
Methyl benzoate	0.733	0.85	0.00	0.46	1.0726	0.35	2.12
Benzyl benzoate	1.264	1.42	0.00	0.51	1.6804	1.49	3.97
Benzonitrile	0.742	1.11	0.00	0.33	0.8711	-0.06	1.56
Aniline	0.955	0.96	0.26	0.50	0.8162	-0.28	0.90
*o*-Toluidine	0.970	0.90	0.23	0.59	0.9751	-0.04	1.32
3-Chloroaniline	1.050	1.10	0.30	0.36	0.9390	0.47	1.88
4-Chloroaniline	1.060	1.10	0.30	0.35	0.9390	0.41	1.84
2-Nitroaniline	1.180	1.37	0.30	0.36	0.9904	0.25	1.79
3-Nitroaniline	1.200	1.71	0.40	0.35	0.9904	0.07	1.32
4-Nitroaniline	1.220	1.91	0.42	0.38	0.9904	0.11	1.39
Nitrobenzene	0.871	1.11	0.00	0.28	0.8906	0.16	1.85
2-Nitroanisole	0.965	1.34	0.00	0.38	1.0902	0.17	1.73
Benzamide	0.990	1.50	0.49	0.67	0.9728	-0.39	0.64
4-Aminobenzamide	1.340	1.94	0.80	0.94	1.0726	-0.86	-0.41
Acetanilide	0.870	1.36	0.46	0.69	1.1137	-0.17	1.19
4-Chloroacetanilide	0.980	1.50	0.64	0.51	1.2357	0.51	2.12
Phenol	0.805	0.89	0.60	0.30	0.7751	0.05	1.48
3-Methylphenol	0.822	0.88	0.57	0.34	0.9160	0.38	2.02
2,3-Dimethylphenol	0.850	0.90	0.52	0.36	1.0569	0.64	-
2,4-Dimethylphenol	0.840	0.80	0.53	0.39	1.0569	0.71	2.42
Thymol	0.822	0.79	0.52	0.44	1.3387	1.20	3.34
4-Chlorophenol	0.915	1.08	0.67	0.20	0.8975	0.79	2.39
Catechol	0.970	1.10	0.88	0.47	0.8338	-0.09	0.88
Resorcinol	0.980	1.00	1.10	0.58	0.8338	-0.18	0.80
Hydroquinone	1.000	1.00	1.16	0.60	0.8338	-0.43	0.58
2-Naphthol	1.520	1.08	0.61	0.40	1.1441	1.04	2.84
1,2,3-Trihydroxybenzene	1.165	1.35	1.35	0.62	0.8925	-0.29	-
Furan	0.369	0.53	0.00	0.13	0.5363	-0.31	1.34
2,3-Benzofuran	0.888	0.83	0.00	0.15	0.9053	0.71	2.67
Quinoline	1.268	0.97	0.00	0.51	1.0443	0.20	2.15
Pyrrole	0.613	0.73	0.41	0.29	0.5774	-0.44	0.75
Pyrimidine	0.606	1.00	0.00	0.65	0.6342	-1.03	-0.40
Antipyrine	1.320	1.50	0.00	1.48	1.5502	-0.67	0.56
Caffeine	1.500	1.60	0.00	1.33	1.3632	-0.77	-0.01
Corticosterone	1.860	3.43	0.40	1.63	2.7389	0.65	1.90
Cortisone	1.960	3.50	0.36	1.87	2.7546	0.23	1.47
Hydrocortisone	2.030	3.49	0.71	1.90	2.7975	0.39	1.53
Estradiol	1.800	3.30	0.88	0.95	2.1988	1.35	4.01
Estratriol	2.000	3.36	1.40	1.22	2.2575	0.67	2.45
Monuron	1.140	1.50	0.47	0.78	1.4768	0.32	1.94
Myrcene	0.483	0.29	0.00	0.21	1.3886	-	4.17
α-Pinene	0.446	0.14	0.00	0.12	1.2574	-	4.83
Geraniol	0.513	0.63	0.39	0.66	1.4903	1.07	-

^a)^From references [16-30].

**Table 2.** Normalized coefficients and *d* distances of the compared systems.

System	*e*_u_	*s*_u_	*a*_u_	*b*_u_	*v*_u_	n	SD	R^2^	*d*	Ref.
TTAB MEEKC	0.13	-0.19	0.06	-0.65	0.72	59	0.12	0.972	-	This work
Octanol-water	0.11	-0.20	0.01	-0.65	0.72	613	0.12	0.994	0.06	[31]
SDS MEEKC	0.07	-0.16	-0.01	-0.67	0.72	53	0.09	0.988	0.11	[3]

**Table 3.** Parameters resulting from the fit of Eq. 17 to log *D*_o/w_ vs pH data. *k*_(BH+)_ and *k*_(B)_ have been experimentally determined in the TTAB-MEEKC system.

Compound	p*K*_a_’ (SD)	log *P*_o/w(BH+)_ (SD)	log *P*_o/w(B)_ (SD)	R^2^	SD	F	*k*_(BH+)_	*k*_(B)_
Alprenolol	9.63 (0.28)	-0.46 (0.16)	3.20 (0.23)	0.939	0.36	116	0.67	11.09
Nadolol	9.31 (0.21)	-2.05 (0.11)	0.83 (0.15)	0.975	0.21	156	0.05	0.88
Oxprenolol	9.38 (0.16)	-1.43 (0.10)	2.14 (0.13)	0.985	0.18	328	0.14	2.82
Penbutolol	9.37 (0.31)	1.23 (0.24)	4.06 (0.24)	0.987	0.24	37	3.20	61.64
Pindolol	9.32 (0.17)	-1.39 (0.10)	1.79 (0.13)	0.970	0.23	243	0.16	2.43
Propranolol	9.24 (0.26)	-0.17 (0.15)	2.99 (0.21)	0.908	0.37	94	1.19	16.51

**Table 4.** Differences between literature (log *D*_lit._) and estimated (log *D*_est_*_._*) values of the bases at different pH values through Eq. 15.

Compound	pH	*α*	log *D*_lit_*_._*^a)^	log *D*_est_*_._*	log *D*_lit_*_._* – log D*_est_*
Alprenolol	2.0	1.00	0.13	1.06	-0.93
	2.0	1.00	-0.89	1.06	-1.95
	3.6	1.00	-0.80	1.06	-1.86
	4.5	1.00	0.14	1.06	-0.92
	4.5	1.00	-0.77	1.06	-1.83
	5.6	1.00	-0.60	1.06	-1.66
	6.6	1.00	0.68	1.07	-0.39
	6.7	1.00	0.73	1.07	-0.34
	7.0	1.00	0.51	1.08	-0.57
	7.4	0.99	1.09	1.12	-0.03
	7.4	0.99	1.08	1.12	-0.04
	8.0	0.98	1.44	1.28	0.16
	8.2	0.96	1.60	1.38	0.22
	8.5	0.93	1.63	1.59	0.04
	9.2	0.73	2.59	2.26	0.33
	10.2	0.21	3.20	2.94	0.26
	11.0	0.04	2.84	3.08	-0.24
	13.0	0.00	3.50	3.11	0.39
Nadolol	2.0	1.00	-2.20	-0.84	-1.36
	3.6	1.00	-2.10	-0.84	-1.26
	4.5	1.00	-2.00	-0.84	-1.16
	5.6	1.00	-1.80	-0.83	-0.97
	6.7	1.00	-1.60	-0.81	-0.79
	7.4	0.99	-1.30	-0.70	-0.60
	7.65	0.98	-0.82	-0.61	-0.21
	8.2	0.93	0.08	-0.26	0.34
	9.2	0.56	0.22	0.70	-0.48
	10.2	0.11	0.76	1.17	-0.41
	13.0	0.00	0.93	1.26	-0.33
Oxprenolol	2.0	1.00	-1.70	-0.08	-1.62
	3.6	1.00	-1.40	-0.08	-1.32
	4.5	1.00	-1.30	-0.08	-1.22
	5.6	1.00	-1.04	-0.08	-0.96
	6.7	1.00	-0.30	-0.06	-0.24
	7.0	1.00	-0.37	-0.03	-0.34
	7.4	0.99	0.13	0.05	0.08
	7.65	0.98	0.69	0.13	0.56
	8.0	0.96	0.61	0.33	0.28
	8.2	0.94	0.76	0.49	0.27
	9.2	0.60	1.67	1.49	0.18
	10.2	0.13	2.10	2.01	0.09
	13.0	0.00	2.16	2.11	0.05
Penbutolol	1.2	1.00	1.24	2.20	-0.96
	7.4	0.99	1.97	2.33	-0.36
	7.65	0.98	2.53	2.41	0.12
	13.0	0.00	4.06	4.36	-0.30
Pindolol	2.0	1.00	-1.17	0.01	-1.18
	2.0	1.00	-1.60	0.01	-1.61
	3.6	1.00	-1.55	0.01	-1.56
	4.5	1.00	-1.40	0.01	-1.41
	4.5	1.00	-1.22	0.01	-1.23
	5.6	1.00	-1.35	0.01	-1.36
	6.6	1.00	-0.56	0.03	-0.59
	6.7	1.00	-0.62	0.04	-0.66
	7.0	1.00	-0.92	0.06	-0.98
	7.4	0.99	0.29	0.13	0.16
	7.4	0.99	-0.10	0.13	-0.23
	8.0	0.95	0.19	0.38	-0.19
	8.2	0.93	0.46	0.52	-0.06
	8.5	0.87	1.18	0.78	0.40
	9.2	0.57	1.29	1.45	-0.16
	10.2	0.12	1.72	1.91	-0.19
	11.0	0.02	1.82	1.98	-0.16
	13.0	0.00	1.80	2.00	-0.20
Propranolol	2.0	1.00	0.20	1.48	-1.28
	2.0	1.00	-0.58	1.48	-2.06
	3.6	1.00	-0.55	1.48	-2.03
	4.5	1.00	0.18	1.48	-1.30
	4.5	1.00	-0.59	1.48	-2.07
	5.5	1.00	0.7	1.5	-0.8
	5.6	1.00	-0.46	1.48	-1.94
	6.5	1.00	0.9	1.50	-0.6
	6.6	1.00	0.68	1.50	-0.82
	6.7	1.00	0.54	1.50	-0.96
	7.0	0.99	0.73	1.53	-0.80
	7.4	0.99	1.4	1.6	-0.2
	7.4	0.99	1.20	1.60	-0.40
	7.4	0.99	1.07	1.60	-0.53
	7.65	0.97	1.62	1.68	-0.06
	8.0	0.95	1.72	1.86	-0.14
	8.2	0.92	1.67	2.01	-0.34
	8.5	0.85	1.60	2.27	-0.67
	9.2	0.52	2.32	2.91	-0.59
	10.2	0.10	2.98	3.33	-0.35
	11.0	0.02	2.94	3.38	-0.44
	13.0	0.00	3.28	3.40	-0.12

^a)^ From references [32–37].
